# Situs Inversus: Inferior-Lateral ST-Elevation Myocardial Infarction on Right-Sided Electrocardiogram

**DOI:** 10.5811/cpcem.2019.5.42912

**Published:** 2019-07-01

**Authors:** Mohamed S. Hamam, Howard Klausner

**Affiliations:** * Henry Ford Health System, Department of Emergency Medicine, Detroit, Michigan; † Henry Ford Health System, Department of Internal Medicine, Detroit, Michigan

## Abstract

Dextrocardia is a rare anatomical anomaly in which the heart is located in the patient’s right hemithorax with its apex directed to the right. Although it usually does not pose any serious health risks, patients with undiagnosed dextrocardia present a diagnostic challenge especially in those presenting with chest pain. Traditional left-sided electrocardiograms (ECG) inadequately capture the electrical activity of a heart positioned in the right hemithorax, which if unnoticed could delay or even miss an acute coronary syndrome diagnosis. Here, we present a case of a patient with dextrocardia presenting with chest pain and diagnosed with ST-elevation myocardial infarction using a right-sided ECG.

## CASE PRESENTATION

**Image 1:**
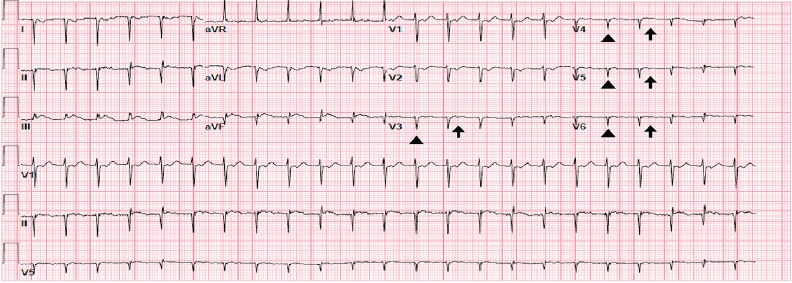
A traditional electrocardiogram in a patient with dextrocardia with poor R-wave progression (arrowheads) and flattened T-waves (arrows). Heart rate: 138 beats per minute. PR interval: 96 milliseconds (ms). QRS interval: 72 ms. QT/QTc: 304/460 ms.

**Image 2:**
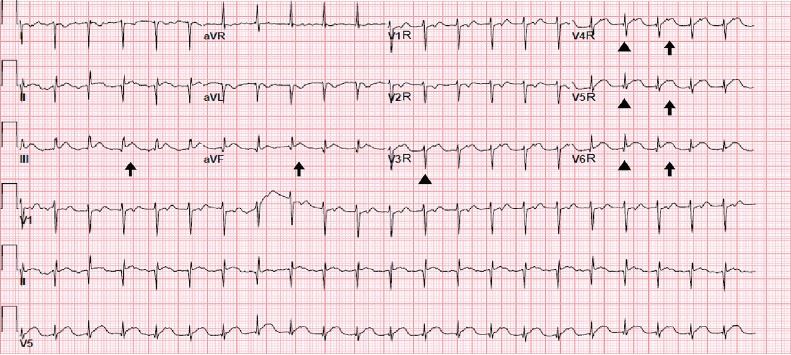
A right-sided electrocardiogram in the same patient with appropriate R-wave progression (arrowheads) and ST elevations (arrows) in the precordial leads. Heart rate: 132 beats per minute. PR interval: 96 milliseconds (ms). QRS interval: 66 ms. QT/QTc: 286/423 ms.

A 52-year-old man presented to the emergency department with two days of intermittent, substernal, crushing chest pain radiating to his right shoulder that woke him from sleep. His medical history included Evan’s syndrome, hypertension, beta-thalassemia, and situs inversus. On physical examination, the patient had a heart rate of 138 beats per minute and a blood pressure of 141/96 millimeters of mercury (mmHg). Initial 12-lead electrocardiogram (ECG) obtained showed poor R-wave progression and flat T-waves in the precordial leads and ST-elevation in inferior leads (Image 1). Given these ECG abnormalities, further history was obtained from the patient who said that a doctor had once told him his heart “faces the wrong way.” Therefore, a right-sided 12-lead ECG was obtained showing ST elevations in V4R, V5R, V6R, III and aVF suggestive of inferior-lateral ischemia (Image 2). The patient was treated with aspirin, heparin and ticagrelor, and cardiac catheterization lab was activated. The patient was found to have an occluded right coronary artery and underwent right coronary artery stenting for obstructive disease. The right-sided 12-lead ECG was essential in detecting lateral ischemia in this patient with dextrocardia.

**CPC-EM Capsule**

What do we already know about this clinical entity?

Dextrocardia is the abnormal location of the heart in the thorax that can cause irregularities in traditional left-sided electrocardiograms (ECG).

What is the major impact of the image(s)?

The images show a left-sided ECG with unclear ST changes and a right-sided ECG showing the full extent of an ST-elevation myocardial infarction in a patient with dextrocardia.

How might this improve emergency medicine practice?

Thorough evaluation of ECGs for appropriate lead placement is important to accurately interpret pathology and life-threatening disease in the emergency department.

## DISCUSSION

Dextrocardia describes an anatomical anomaly in which the heart is located in the right hemithorax with its apex directed to the right. However, the anatomical location of the atria, ventricles and great vessels vary depending on the embryological development of the heart.^1^ In situs inversus, also called mirror-image dextrocardia, the great vessels and chambers are positioned as a mirror image of a left-sided heart.^1^ This malpositioning produces interesting abnormalities that can be seen on a traditional ECG with poor R-wave progression and lack of T-waves.^1^ Without noticing these details, a devastating diagnosis such as ST-elevation myocardial infarction could potentially be missed. It is, therefore, always important to scrutinize all ECGs and address any abnormality that could not be explained anatomically. Quick changes to the positioning of the ECG leads provide a depth of information that could have otherwise been missed.
